# A Novel Dye-Modified Metal–Organic Framework as a Bifunctional Fluorescent Probe for Visual Sensing for Styrene and Temperature

**DOI:** 10.3390/molecules28134919

**Published:** 2023-06-22

**Authors:** Jie Yang, Chaojun Ren, Min Liu, Wenwei Li, Daojiang Gao, Hongda Li, Zhanglei Ning

**Affiliations:** 1 College of Chemistry and Materials Science, Sichuan Normal University, Chengdu 610068, China; yangjie_deyouxiang@163.com (J.Y.); liumin200009@163.com (M.L.); liwenwen202202@163.com (W.L.); daojianggao@sicnu.edu.cn (D.G.); 2 Beijing Aerospace Propulsion Institute, Beijing 100076, China; rcj_ustb@163.com; 3 Liuzhou Key Laboratory for New Energy Vehicle Power Lithium Battery, School of Electronic Engineering, Guangxi University of Science and Technology, Liuzhou 545006, China; hdli@gxust.edu.cn

**Keywords:** MOFs, fluorescent probe, dye, styrene, temperature sensing

## Abstract

A novel fluorescent probe (C460@Tb-MOFs) was designed and synthesized by encapsulating the fluorescent dye 7-diethylamino-4-methyl coumarin (C460) into a terbium-based metal–organic framework using a simple ultrasonic impregnation method. It is impressive that this dye-modified metal–organic framework can specifically detect styrene and temperature upon luminescence quenching. The sensing platform of this material exhibits great selectivity, fast response, and good cyclability toward styrene detection. It is worth mentioning that the sensing process undergoes a distinct color change from blue to colorless, providing conditions for the accurate visual detection of styrene liquid and gas. The significant fluorescence quenching mechanism of styrene toward C460@Tb-MOFs is explored in detail. Moreover, the dye-modified metal–organic framework can also achieve temperature sensing from 298 to 498 K with high relative sensitivity at 498 K. The preparation of functionalized MOF composites with fluorescent dyes provides an effective strategy for the construction of sensors for multifunctional applications.

## 1. Introduction

Metal–organic framework (MOF) materials have attracted much attention due to their porosity, large specific surface area, versatility, and tunable functionality [1,2]. As typical porous materials, MOFs can be used as a unique tool to stabilize and limit functional substances, which enables the development of diverse MOF composites and their application in different fields [3,4,5]. In particular, the encapsulation of fluorescent dye into the pore spaces of MOFs greatly reduces the aggregation-induced quenching effect of dyes, which does not change the original structure of MOFs [6]. In addition, the dye still has good photochemical stability due to the protection of the MOF. The dye-modified metal–organic framework possesses dual fluorescent groups from the MOFs and dyes, extending the range of applications to cell staining, fluorescence immunization, and fluorescent probes [7,8,9,10].

Volatile organic compounds (VOCs) are generally defined as organic compounds with a saturated vapor pressure above 133.32 Pa at room temperature and a boiling point below 250 °C at an atmospheric pressure of 100 kPa [11,12]. When VOCs are present in the environment at certain concentrations, they can have a significant impact on human health. Irritating odors can cause fatigue, headaches, nausea, and vomiting in the short term, and even cause adverse effects such as coma and convulsions. In addition, long-term exposure to VOCs can have even more detrimental effects on the human body, including damage to the kidneys, liver, central nervous system, and even cancer [13,14]. Styrene, a typical component of VOCs, is widely used in the synthetic resin, pharmaceutical, dye, pesticide, and mineral processing industries [15,16,17], and the global total production capacity of styrene has exceeded 36 million tons in recent years. Styrene is classified as a Group 2B carcinogen by the World Health Organization’s International Agency for Research on Cancer, and it can be absorbed by the body through the respiratory tract and skin [18]. Several methods have been reported for the detection of VOCs, mainly gas chromatography, thermal desorption mass spectrometry, Fourier transform infrared spectrometry, atomic emission spectrometry, and semiconductor electrochemistry [19,20,21,22]. However, the above detection methods have some inevitable problems, such as complex operational processes, long detection times, and high technical requirements, all of which can limit widespread detection. Therefore, developing a simple, time-saving, and low-cost method to accurately measure styrene is of great importance.

Temperature is a fundamental physical parameter of great importance in human life, scientific research, and industry. Temperature is not only a key factor in the growth of plants and animals, but it also plays a crucial role in the fields of optics, electrochemistry, and biomedicine [23,24]. Early thermometers were contact thermometers, which often measured temperature by changes in volume, potential, and conductance, but they were less suitable in certain special environments (liquids, cells, or inside the body, etc.). Luminescent thermometers have received much attention in the field of non-contact optical temperature measurement due to their simplicity, high sensitivity, and accuracy, and the temperature dependence of fluorescence is used as an indicator of temperature sensing. In recent years, many composite MOF materials with a fast response and high relative sensitivity have been explored and developed for use as luminescent thermometers [25,26].

In this work, a dye C460-modified C460@Tb-MOF composite was synthesized. It can be applied as a fluorescent probe for the visual recognition of styrene liquid and gas, exhibiting high sensitivity and a fast response rate. The quenching mechanism of styrene by the C460@Tb-MOF composite is also explored. The portable C460@Tb-MOF luminescent silica gel plate was prepared to obtain a more visual inspection of styrene detection. Moreover, the C460@Tb-MOF composite can be used as a fluorescence probe for temperature sensing from 298 to 498 K. Overall, this work provides a simple strategy for multifunctional MOFs and opens the way for versatile applications in MOF composites.

## 2. Experimental Section

### 2.1. Reagents and Instruments

All chemical reagents and solvents used in this work are commercially available analytical grade and are used directly without further purification. Terbium nitrate hexahydrate (Tb(NO_3_)_3_·6H_2_O), mucic acid (MA), and potassium hydroxide (KOH) were obtained from Aladdin Chemistry Co., Ltd. (Shanghai, China). Benzene, toluene, ethylbenzene, o-xylene, formaldehyde, acetaldehyde, propionaldehyde, butyl acetate, and styrene were purchased from Chengdu Kelong Chemical Co., Ltd. (Chengdu, China).

### 2.2. Synthesis of Tb-MOFs and C460@Tb-MOFs

The Tb-MOF sample is synthesized by a fast and facile method in room temperature conditions [27,28]. The C460@Tb-MOF composites were synthesized by simple ultrasonic impregnation method. First, the Tb-MOFs were immersed in an ethanolic solution of 60 mM C460; the mixture was shaken uniformly by sonication and kept in equilibrium for 30 min, and then immersed at room temperature for 24 h. Finally, the resulting precipitate was collected and dried at 60 °C for 24 h to obtain a yellow-green solid, which was finely ground and then sealed in a dry environment for storage.

### 2.3. Fluorescence Sensing of Styrene Liquid

C460@Tb-MOFs were dissolved in ethanol solution and sonicated to form a homogeneous suspension. A series of 10^−3^ M solutions of VOCs (benzene, toluene, ethylbenzene, o-xylene, formaldehyde, acetaldehyde, propionaldehyde, butyl acetate, and styrene) were then added to the suspension and tested for fluorescence.

### 2.4. Fluorescence Sensing of Styrene Gas

Sodium carboxymethyl cellulose and silica gel were dispersed into an aqueous solution to obtain a uniform mixture of solution, and then coated on a glass slide and left to dry to obtain a matrix silica gel plate. The C460@Tb-MOFs were evenly dispersed in the PVA solution, and then they were evenly dropped on the silica gel plate. The C460@Tb-MOF luminous silica gel plate was prepared by constant temperature drying (60 ℃), and it was used to detect VOC gas.

The prepared C460@Tb-MOF luminous silica gel plate was put into the quartz cuphor, then 20 μL VOCs was added, and the lid of the quartz cuphor was covered to form a closed space full of the gas atmosphere of VOCs. After a certain time of contact, the emission spectrum of the luminous silica gel plate was recorded.

## 3. Results and Discussion

### 3.1. Characterization of C460@Tb-MOF Fluorescent Probe

**Figure 1:**
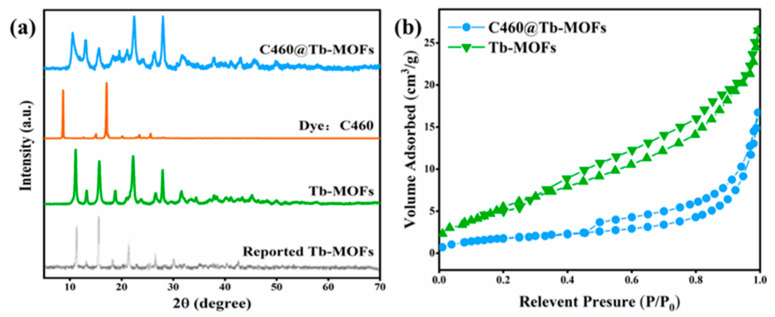
(**a**) XRD patterns of the reported Tb-MOFs, the synthetic crystalline Tb-MOFs, dye C460, and C460@Tb-MOF samples. (**b**) The N_2_ adsorption isotherms of Tb-MOFs and C460@Tb-MOFs after heat treatment.

Figure 1a shows the XRD of the reported Tb-MOFs (the diagram of the 2D network of Tb-MOFs is shown in Figure S1), the synthetic Tb-MOFs, the dye C460, and the C460@Tb-MOFs, respectively. The XRD of the synthesized Tb-MOFs is in a pure phase, which is consistent with diffraction peak positions of the reported Tb-MOFs. In addition, the diffraction peaks of C460@Tb-MOFs with C460 functionalized modification are in the same position as Tb-MOFs, and the crystalline structure is not disrupted [29]. The EDX results of Tb-MOFs and C460@Tb-MOFs are shown in Figure S2. The Tb-MOFs contain three elements: C, O, and Tb, while the C460@Tb-MOFs contain four elements: C, O, N, and Tb, with the same chemical composition as the elemental composition of the target sample. The IR spectra of Tb-MOFs and C460@Tb-MOFs are shown in Figure S3a. The broad band at 3306 cm^−1^ for Tb-MOFs is the -OH vibrational peak of the water molecule, and the strong peak at 3426 cm^−1^ for C460@Tb-MOFs is probably the O-H and N-H stretching vibrations. N2 adsorption tests on Tb-MOFs and C460@Tb-MOFs are shown in Figure 1b. The specific surface area and pore volume of Tb-MOFs are 18.4238 m^2^/g and 0.0406 cm^3^/g, respectively, and those of C460@Tb-MOFs are 6.8437 m^2^/g and 0.0256 cm^3^/g, respectively, which are reduced by 62.9% and 36.9% compared with Tb-MOFs. The surface area and pore volume of the Tb-MOFs are significantly decreased upon loading with C460, indicating that the dye C460 has been introduced into the Tb-MOF’s channel or surface. The zeta potentials of C460, Tb-MOFs, and C460@Tb-MOFs are shown in Figure S4a. It can be seen from Figure S4b that all the surface potentials are negative, and the absolute zeta potential value of C460@Tb-MOFs increases to 38.5 mV. The results show that there is an electrostatic attraction (H-H or N-H interaction) between C460 and Tb-MOFs, which promotes the emission of C460 in C460@Tb-MOFs through host–guest energy transfer, and that the system is more stable with a high absolute values of the zeta potential for C460@Tb-MOFs [30]. Thermal stability and pyrolysis properties are two of the important properties of the materials. The thermogravimetric curves of Tb-MOFs and C460@Tb-MOFs are shown in Figure S3b; both Tb-MOFs and C460@Tb-MOFs have three main stages of weight loss, and in the high temperature range of 400–600 °C, the weight loss rate of C460@Tb-MOFs (14.4%) is smaller than that of Tb-MOFs (24.6%) [31]. The pyrolysis curves of Tb-MOFs and C460@Tb-MOFs are shown in Figure S3c. The difference between the first thermal cracking temperature of the two samples was not significant, and by comparing the second thermal cracking temperature of C460@Tb-MOFs, it increased by 31.8 °C, which indicates that C460@Tb-MOFs have good thermal stability and high temperature resistance to pyrolysis. The morphological characteristics of the Tb-MOFs and C460@Tb-MOFs are shown in Figure S5. All the samples show a large number of small spheres that are 3–5 μm in diameter (Figure S5a,c), indicating that the introduction of C460 had no major effect on the microstructure of Tb-MOFs (also confirmed by XRD). Compared with Tb-MOFs, the SEM image of C460@Tb-MOFs displayed a rough surface (Figure S5b,d), which may be caused by dye C460 adhering to the surface of MOFs. In elemental mapping, four elements O, C, Tb, and N (derived from dye C460) were evenly distributed in C460@Tb-MOF composites (Figure S6), further indicating the successful synthesis of C460@Tb-MOFs.

In order to accurately determine the content of dye C460 in C460@Tb-MOFs, the luminescence intensity of different concentrations of C460 in ethanol was measured. It can be seen that the intensity of materials gradually enhanced as the concentration of C460 increased (Figure S7a), and the linearly fitted concentration and luminescence intensity results are shown in Figure S7b. The resulting relationship equation is as follows: I = 1.2180 × 10^8^ C + 43.6873 (where I is the luminous intensity; C is the concentration of dye C460). The emission spectra of the actual loading of dye C460 in C460@Tb-MOFs are shown in Figure S8a,d, and the actual loading of C460 in different concentrations of C460@Tb-MOFs are calculated by bringing the following into the equation: (a) 1 × 10^−3^ M, 0.004%; (b) 1 × 10^−2^ M, 0.034%; (c) 6 × 10^−2^ M, 0.49%; (d) 1 × 10^−1^ M, 0.67%. The luminescence intensity of C460@Tb-MOFs can be modulated by changes in dye concentration. Given that C460@Tb-MOFs are used as ratio-metric fluorescent probes, a sample with a C460 concentration of 6 × 10^−2^ M and I_545_/I_450_ of 2.0, with an actual loading of 0.49% (Figure S8c), was selected for subsequent application studies. The emission spectra of C460@Tb-MOFs at different excitation wavelengths are shown in Figure S9a. A change in the excitation wavelength leads to a change in the characteristic emission ratio of Tb^3+^/C460. A total of 225 nm was chosen as the excitation wavelength for the sample with the best luminescence and emission intensity ratio. The fluorescence emission spectra of C460@Tb-MOFs after immersion in stable solutions at different pH values (pH = 3.0–9.0) for 48 h are shown in Figure S9b, indicating that the samples have good fluorescence stability, laying the foundation for the subsequent practical application of this material. The excitation and emission spectra of Tb-MOFs and C460 are shown in Figure S10a,b; the excitation and emission spectra of C460@Tb-MOFs are shown in Figure S10c,d. As shown in Figure S10c, the characteristic peak was used as the monitoring wavelength with peaks at 225 nm and 368 nm and 225 nm was chosen as the excitation wavelength in conjunction with Figure S9a. As shown in Figure S10d, when C460@Tb-MOFs were excited at 225 nm, double emission peaks appeared at 450 and 545 nm [32,33]. The samples were yellow-green in daylight and the chromaticity coordinates were (0.175, 0.207) between C460 and Tb^3+^, indicating that C460@Tb-MOFs were successfully prepared and are consistent with the blue-green double emission characteristics of C460/Tb^3+^. The C460 emission peak showed a slight red shift (from 445 to 450 nm), which may be due to the enhanced molecular interactions caused by the loading of C460 on the Tb-MOFs.

### 3.2. C460@Tb-MOFs for the Real-Time Detection of Styrene Liquid and Styrene Gas

**Figure 2:**
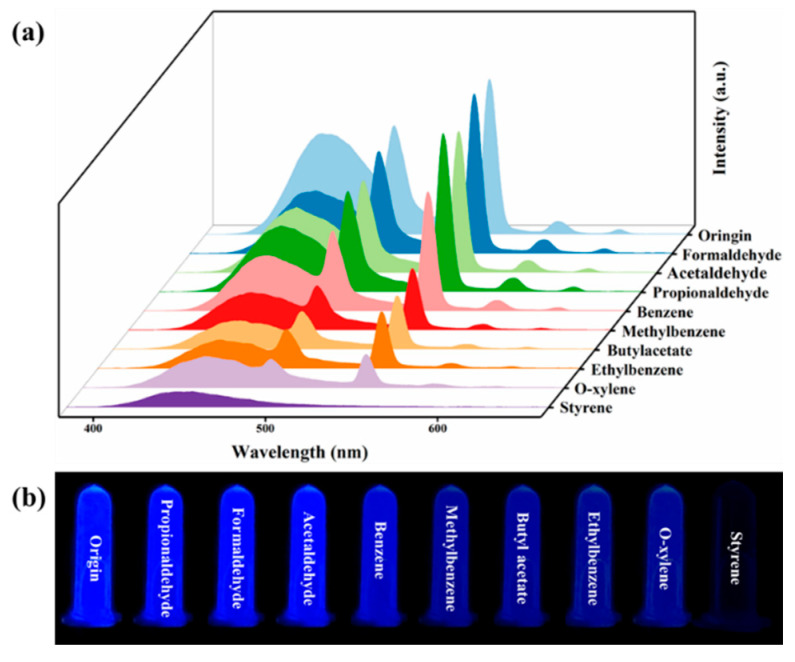
(**a**) Emission spectra of C460@Tb-MOFs dispersed in different VOC liquids. (**b**) Photographs of C460@Tb-MOFs immersed in different VOC liquids under 254 nm UV dark box irradiation.

To investigate the ability of C460@Tb-MOFs as fluorescent probes for the detection of volatile organic compounds (VOCs), a series of VOC solutions (propionaldehyde, formaldehyde, acetaldehyde, benzene, toluene, butyl acetate, ethylbenzene, o-xylene, and styrene) were added to C460@Tb-MOFs to obtain a mixture and tested for their fluorescence response. As shown in Figure 2a, the C460@Tb-MOFs have different fluorescence responses to different VOC liquids. The styrene in the VOC liquid causes a significant decrease in the intensity of the C460 characteristic peak, and the characteristic emission of Tb^3+^ is almost completely quenched by styrene, indicating that C460@Tb-MOFs have excellent detection selectivity for styrene. Figure 2b shows the results of the sample testing VOC liquid under UV light (254 nm), where the fluorescent color of styrene is significantly quenched and can be directly distinguished by the naked eye. The fluorescence quenching rates of C460@Tb-MOFs for different VOC solutions are shown in Figure S11a. The fluorescence quenching rates for other VOCs were below 80% at 450 nm, while the maximum quenching rate for styrene exceeded 80% (85.7%). The quenching rate for styrene was as high as 99.1% at 545 nm, as shown in Figure S11b, which was almost completely quenched. In summary, the C460@Tb-MOFs utilized the double emission feature to selectively detect styrene in VOC solutions. One of the characteristics of an excellent fluorescent probe is the high selectivity that can be achieved even against complex backgrounds. Anti-interference tests were carried out for styrene, as shown in Figure S12. The fluorescence was almost completely quenched by the addition of styrene, which is consistent with the detection of styrene. This result demonstrates the excellent anti-interference capability of the fluorescent probe.

**Figure 3:**
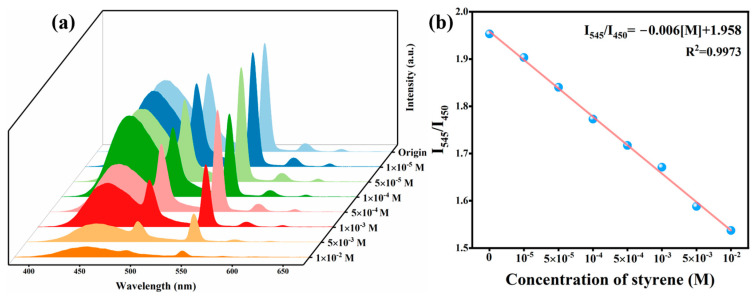
(**a**) Emission spectra after the addition of different styrene concentrations. (**b**) Linear plot of styrene concentration against I_545_/I_450_.

The detection sensitivity of C460@Tb-MOFs for styrene liquids is shown in Figure 3a. The bimodal intensity gradually decreased as the concentration of styrene increase. Meanwhile, the blue fluorescence gradually decreased until it almost disappeared. Fitting the concentration and intensity ratio (I_545_/I_450_), as shown in Figure 3b, there was a good linear relationship for styrene identification in the range of 10^−5^~10^−2^ M: I_545_/I_450_ = −0.006 [M] + 1.958 (R^2^ = 0.9973), indicating that C460@Tb-MOFs can be used as a fluorescent probe for the quantitative detection of styrene liquids [34]. The rapid dropwise addition of styrene liquid to a cuvette of C460@Tb-MOFs suspension for real-time sensing monitoring is shown in Figure S13a. Within less than 5 s of adding styrene liquid, the bimodal intensity immediately dropped to almost total quenching and remained relatively stable in subsequent quenching, indicating a rapid and time-stable response of C460@Tb-MOFs for detecting styrene liquid. Figure S13b shows the time dependence of I_545_/I_450_, visually reflecting the significant detection effect after 5 s of styrene addition and the relative stability in 5-600 s. These results indicate that the C460@Tb-MOFs have a rapid response to styrene detection, showing that they are more advantageous for practical applications.

**Figure 4:**
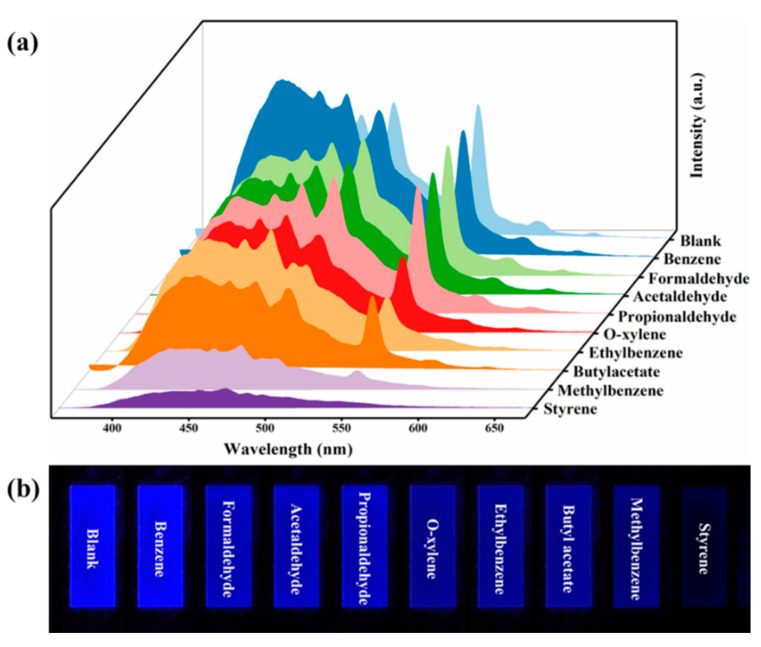
(**a**) Emission spectra of C460@Tb-MOFs in different VOC vapor atmospheres. (**b**) The photographs of C460@Tb-MOFs (after being kept in various VOC vapor atmospheres for a period of times) under 254 nm UV light irradiation.

Given the extremely volatile nature of VOCs, the possibility of C460@Tb-MOFs being used as a fluorescent probe for styrene gas continues to be explored. Portable C460@Tb-MOF luminescent silica gel plates were prepared to test VOC gases, as shown in Figure 4a. The detection of different VOC gases varied, but the most significant quenching effect was for styrene (I_545_). The luminescent silica gel plates kept in the VOC gas atmosphere and photographed under the UV dark box (254 nm) are shown in Figure 4b. The luminescent silica gel plates exhibit a superb quenching effect of the luminescent silica gel plate on styrene gas, showing excellent selectivity and visual sensing for styrene gas.

The fluorescence quenching rates of C460@Tb-MOF luminescent silica gel plates for different VOC gases at 450 nm and 545 nm are shown in Figure S14a,b. The identification of different VOC gases differed, but the quenching rate for styrene gas was over 80% (87.5% for I_450_ and 95.0% for I_545_); therefore, C460@Tb-MOF luminescent silica gel plates can be used as specific detectors for the gas styrene. The results of the temporal response of the C460@Tb-MOF luminescent silica gel plate to styrene gas detection are shown in Figure S15a. The intensity of I_545_ decreased significantly within 10 s and was almost completely quenched after 1 min, while the intensity of I_450_ decreased by about 1/3 within 10 s, decreased significantly, and remained relatively stable after 1 min. The relative intensity ratio versus time is shown in Figure S15b. It can be found that the intensity ratio changed significantly within 10 s of detection, indicating that this C460@Tb-MOF luminescent silica gel plate is capable of the rapid detection of styrene gas. Moreover, the cycling performance of the C460@Tb-MOF luminescent silica gel plate for styrene gas detection was investigated, and the styrene gas was dried and treated before the next detection. As shown in Figure S16, the relative fluorescence intensity ratio remained at the initial value for five cycles. The C460@Tb-MOF luminescent silica gel plate has a good cycling performance for styrene gas detection and is expected to be widely used in practical environments due to its outstanding advantages, such as portability and cyclability.

### 3.3. C460@Tb-MOF Sensing Mechanism for Styrene

**Figure 5:**
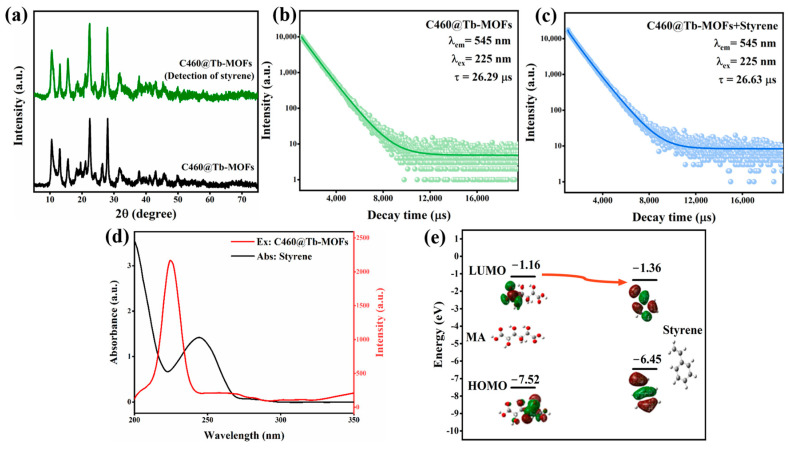
(**a**) XRD patterns of C460@Tb-MOFs and C460@Tb-MOFs after detection of styrene; (**b**,**c**) fluorescence lifetime of C460@Tb-MOFs before and after detection of styrene; (**d**) excitation spectra of C460@Tb-MOFs and UV absorption spectra of styrene; (**e**) the calculated LOMO and HUMO energy levels of ligand mucic acid (MA) and styrene.

The mechanism of fluorescence quenching can be attributed to the following: the structural disintegration of the material; interactions with rare earth ions; and interactions with ligands. The possible fluorescence quench mechanisms for the detection of styrene are therefore investigated. The XRD of C460@Tb-MOFs after the detection of styrene was first tested as shown in Figure 5a, and the diffraction peak position did not change when comparing C460@Tb-MOFs, thus ruling out fluorescence quenching due to the structural disintegration of the material.

The fluorescence quenching mechanism can also be verified by the fluorescence decay lifetime, and the fluorescence lifetime at 545 nm, with the maximum quenching rate, was selected for exploration. As shown in Figure 5b,c, the fluorescence lifetimes of C460@Tb-MOFs did not change significantly before and after the detection of styrene (before: 26.29 μs; after: 26.63 μs), which is a static quenching process. Since the styrene molecule has no functional groups coordinated to Tb^3+^, it is likely that the interaction is with the ligand. Exploring the possible quenching mechanism further, the excitation spectra of C460@Tb-MOFs and the UV absorption spectra of styrene are shown in Figure 5d. The apparent overlap in the shortwave region leads to fluorescence resonance energy transfer, where the ligand energy is transferred to and absorbed by styrene, reducing the Tb^3+^ “antenna effect”, and fluorescence quenching is consistent with a FRET mechanism. The LOMO and HUMO energy levels of the ligand (MA) and styrene molecules were calculated using the density functional theory, as shown in Figure 5e. The LUMO energy level of the acceptor styrene (−1.36 eV) is lower than that of the donor MA (−1.16 eV), while the LUMO-HOMO band gap value of styrene (5.09 eV) is lower than that of MA (6.36 eV), so MA as a donor can transfer energy to the acceptor styrene, leading to fluorescence quenching of C460@Tb-MOFs, which is also consistent with the above fluorescence measurements. This suggests that the detection of styrene fluorescence quenching is attributed to a PET mechanism. In summary, the mechanism of styrene detection by fluorescent probes of C460@Tb-MOFs is mainly fluorescence resonance energy transfer (FRET) and photo-induced electron transfer (PET) between styrene and C460@Tb-MOFs.

### 3.4. C460@Tb-MOFs for Temperature Sensing

**Figure 6:**
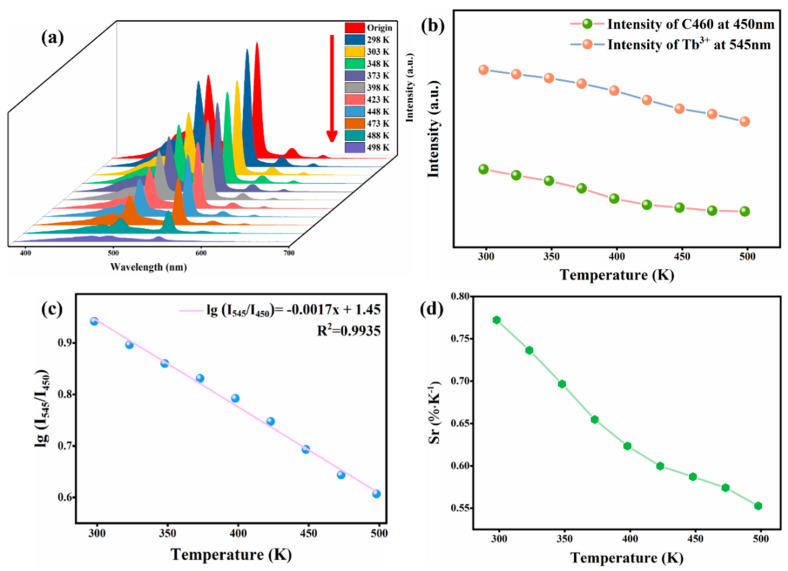
(**a**) Emission spectra of C460@Tb-MOFs in different temperature ranges (298~498 K). (**b**) Trends of fluorescence intensity with temperature at 450 nm and 545 nm for C460@Tb-MOFs, respectively. (**c**) Linear fits of temperature (298~498 K) related to the intensity ratio of I_545_/I_450_. (**d**) Relative sensitivity of C460@ Tb-MOFs with temperature-dependent relative sensitivity.

In view of the outstanding fluorescence properties of C460@Tb-MOFs, other properties of this material were investigated. C460@Tb-MOFs were used as fluorescent probes for temperature sensing, as shown in Figure 6a. Fluorescence properties were tested in 298~498 K, with increasing temperatures, the characteristic peak of C460@Tb-MOFs gradually decreased with good temperature dependence. Figure 6b shows that the fluorescence intensity of C460 and Tb^3+^ decreased regularly with increasing temperatures in 298~498 K. The relationship between C460@Tb-MOFs and temperature was further explored, and the results of a linear fit of temperature versus I_545_/I_450_ are shown in Figure 6c. From the graph, there is a functional relationship between the temperature and I_545_/I_450_ with the fitted function equation lg(I_545_/I_450_) = −0.0017*x* + 1.45 with a linear correlation coefficient (R^2^) of 0.9935, indicating that the composite can act as a ratio-metric fluorescent temperature probe in 298-498 K. In addition, differences in the temperature performance of fluorescent temperature probes can be compared by the relative sensitivity (*S*_r_), which is defined as self-calibrating luminescence thermometry, as shown below:

$$S_{r}=\left|\frac{\partial Y/\partial T}{Y}\right|$$

In the above equation *S*_r_ is the relative sensitivity, *Y* is the lg(I_545_/I_450_) intensity ratio, and *T* is the temperature. The calculated relative sensitivity results in 298~498 K are shown in Figure 6d, where *S*_r_ for C460@Tb-MOFs exhibits a regular variation, with *S*_r_ at 498 K still maintaining 0.55% K^−1^, which is better than *S*_r_ at high temperatures for other materials (Table 1).

## 4. Conclusions

In summary, we proposed a facile strategy to obtain a novel blue-green dual-emitting functionalized C460@Tb-MOF composite by simply introducing dye into Tb-MOFs. The experimental results show that the developed C460@Tb-MOFs can be used as an outstanding fluorescent probe for the specific detection of styrene liquids with great selectivity, fast response, and high sensitivity. In addition, the home-made luminescent silica gel plates can directly discriminate styrene gas with the naked eye with excellent cycling performance. The quenching mechanism can be a combination of FRET and PET rules. Moreover, C460@Tb-MOFs can also respond well as a temperature probe to achieve temperature sensing at 298~498 K with high relative sensitivity at high temperatures. To the best of our knowledge, this study realizes styrene detection and temperature sensing simultaneously for the first time, which successfully extends the application of metal–organic frameworks in the field of fluorescence recognition.

## Figures and Tables

**Table 1:** Comparison of relative sensitivity of luminescent MOFs in different temperature ranges.

MOFs	Temperature Range (K)	*S*_r_ (% K^−1^)	Max Temperature (K)	Ref
Rh101@UiO-67	293~333	1.19	333	[35]
Tb_0.99_Eu_0.01_(BDC)_1.5_(H_2_O)_2_	300~320	0.37	320	[36]
Cdots&RB@ZIF-8	293~353	0.74	353	[24]
Eu@Uio-(bpydc)	293~353	0.31	353	[25]
Dycpia	298~473	0.42	473	[26]
[Eu_0.7_Tb_0.3_(cam)(Himdc)_2_(H_2_O)_2_]_3_	100~450	0.079	450	[37]
[Tb_0.99_Eu_0.01_(hfa)_3_(dpbp)]_n_	200~300	0.52	300	[38]
Tb_0.9_Eu_0.1_L	40~300	0.11	300	[39]
[(Tb_0.9382_Eu_0.0616_)(bpcd)_2_(NO_3_)_2_]Cl	25~200	0.34	200	[40]
Tb_0.92_Eu_0.08_-HPIDC-OX	303~473	0.36	473	[23]
C460@Tb-MOFs	298~498	0.55	498	this work

## Data Availability

Not applicable.

## Supplementary Materials

The following supporting information can be downloaded at: https://www.mdpi.com/article/10.3390/molecules28134919/s1, Figure S1 The diagram of the 2D network of Tb-MOFs; Figure S2 EDX spectrum of the as-obtained C460@Tb- UiO-66-(COOH)_2_; Figure S3 (a) FT-IR spectra of Tb-MOFs and C460@ Tb-MOFs; (b) The thermogravimetric curve of Tb-MOFs and C460@Tb-MOFs; (c) The DTG curve of Tb-MOFs and C460@Tb-MOFs; Figure S4 (a) Zeta potential plots of C460, Tb-MOFs and C460@Tb-MOFs; (b) Histograms of zeta potential values of C460, Tb-MOFs and C460@Tb-MOFs; Figure S5 (a) SEM image of Tb-MOFs (low magnification); (b) SEM image of Tb-MOFs (high magnification), inset shows the details at this magnification; (c) SEM image of C460@Tb-MOFs (low magnification); (d) SEM image of C460@Tb-MOFs (high magnification), inset shows the details at this magnification; Figure S6 Elemental mapping images of C460@Tb-MOFs: (a) O element; (b) C element; (c) Tb element; (d) N element; Figure S7 (a) Emission spectra of concentration gradient dye C460; (b) Fitted relationship between C460 luminescence intensity and concentration; Figure S8 Emission spectra of C460@Tb-MOFs (different concentrations of dye): (a) The concentrations of C460 is 10-3 M with 0.004% loading; (b) The concentrations of C460 is 10-2 M with 0.034% loading; (c) The concentrations of C460 is 6×10-2 M with 0.49% loading; (d) The concentrations of C460 is 10-1 M with 0.67% loading; Figure S9 (a) Emission spectra of C460@Tb-MOFs at different excitation wavelengths; (b) Emission spectra of C460@Tb-MOFs in different pH environments; Figure S10 (a) The excitation and emission spectra of Tb-MOFs, inset is CIE chromaticity diagram for the Tb-MOFs; (b) The excitation and emission spectra of C460, inset is CIE chromaticity diagram for the C460; (c) Excitation spectra of C460@Tb-MOFs; (d) Emission spectra of C460@Tb-MOFs, inset is CIE chromaticity diagram for the C460@Tb-MOFs; Figure S11 (a) The quenching rates of C460@Tb-MOFs at I450 for different VOCs solutions; (b) The quenching rates of C460@Tb-MOFs at I545 for different VOCs solutions; Figure S12 Anti-interference of C460@Tb-MOFs to styrene identification in the context of the presence of other interfering VOCs; Figure S13 (a) Emission spectra of the time response of styrene detection by C460@Tb-MOFs; (b) Relative intensity ratio (I545/I450) versus time for styrene detection by C460@Tb-MOFs; Figure S14 (a) The quenching rates of C460@Tb-MOFs at I450 for different VOCs gases; (b) The quenching rates of C460@Tb-MOFs at I545 for different VOCs gases; Figure S15 (a) Emission spectra of the time response of C460@Tb-MOFs silica gel plates for the detection of styrene gas; (b) The relative intensity ratio versus time for the detection of styrene gas by C460@Tb-MOFs silica gel plates; Figure S16 The histogram of relative fluorescence intensity of C460@Tb-MOFs silica gel plate after five cycles of styrene gas detection.

## References

[1] Li Q., Chen H., You S., Lin Z., Chen Z., Huang F., Qiu B. (2023). Colorimetric and fluorescent dual-modality sensing platform based on UiO-66 for fluorion detection. Microchem. J..

[2] Liu M., Ren X., Meng X., Li H. (2021). Metal-organic frameworks-based fluorescent nanocomposites for bioimaging in living cells and in vivo. Chin. J. Chem..

[3] Cai H., Lu W., Yang C., Zhang M., Li M., Che C.-M., Li D. (2019). Tandem Förster resonance energy transfer induced luminescent ratiometric thermometry in Dye-encapsulated biological metal–organic frameworks. Adv. Opt. Mater..

[4] Ruan B., Yang J., Zhang Y.-J., Ma N., Shi D., Jiang T., Tsai F.-C. (2020). UiO-66 derivate as a fluorescent probe for Fe^3+^ detection. Talanta.

[5] Fan M., Yan J., Cui Q., Shang R., Zuo Q., Gong L., Zhang W. (2023). Synthesis and Peroxide Activation Mechanism of Bimetallic MOF for Water Contaminant Degradation: A Review. Molecules.

[6] Fan Y., Jiang X., Che J., Li M., Zhang X., Gao D., Bi J., Ning Z. (2022). A Ratiometric Fluorescent Sensor Based on Dye/Tb (III) Functionalized UiO-66 for Highly Sensitive Detection of TDGA. Molecules.

[7] Liu J., Yue X., Wang Z., Zhang X., Xu Y. (2020). Coumarin 7 functionalized europium-based metal–organic-framework luminescent composites for dual-mode optical thermometry. J. Mater. Chem. C.

[8] Liu L., Lu X.-Y., Zhang M.-L., Ren Y.-X., Wang J.-J., Yang X.-G. (2022). 2D MOF nanosheets as an artificial light-harvesting system with enhanced photoelectric switching performance. Inorg Chem Front..

[9] Wang X., Wang X., Han Y., Li H., Kang Q., Wang P., Zhou F. (2019). Immunoassay for cardiac troponin I with fluorescent signal amplification by hydrolyzed coumarin released from a metal–organic framework. ACS Appl. Energy Mater..

[10] Feng D., Zhang T., Zhong T., Zhang C., Tian Y., Wang G. (2021). Coumarin-embedded MOF UiO-66 as a selective and sensitive fluorescent sensor for the recognition and detection of Fe^3+^ ions. J. Mater. Chem. C.

[11] Shen Y., Tissot A., Serre C. (2022). Recent progress on MOF-based optical sensors for VOC sensing. Chem. Sci..

[12] Li X., Zhang L., Yang Z., Wang P., Yan Y., Ran J. (2020). Adsorption materials for volatile organic compounds (VOCs) and the key factors for VOCs adsorption process: A review. Sep. Purif. Technol..

[13] Tung T.T., Tran M.T., Feller J.-F., Castro M., Van Ngo T., Hassan K., Nine M.J., Losic D. (2020). Graphene and metal organic frameworks (MOFs) hybridization for tunable chemoresistive sensors for detection of volatile organic compounds (VOCs) biomarkers. Carbon.

[14] Joshi M., Nair S. (2008). Hplc analysis of trigonella foenum-graecum seeds to assess phytoestrogens. Indian J. Occup. Environ. Med..

[15] Kim S.-I., Kim A.-R., Bae H.J., Kim S.-Y., Ravi S., Kim K.C., Bae Y.-S. (2021). Cu(I)-incorporation strategy for developing styrene selective adsorbents. Chem. Eng. J..

[16] Yang F., Ma J., Zhu Q., Ma Z., Wang J. (2022). Aggregation-induced luminescence based UiO-66: Highly selective fast-response styrene detection. ACS Appl. Mater. Interfaces.

[17] Kim S.-I., Kim A.-R., Kim S.-Y., Lee J.-Y., Bae Y.-S. (2020). High styrene/ethylbenzene selectivity in a metal-organic framework with coordinatively unsaturated cobalt(II) sites. Sep. Purif. Technol..

[18] Sarigiannis D.A., Karakitsios S.P., Gotti A., Liakos I.L., Katsoyiannis A. (2011). Exposure to major volatile organic compounds and carbonyls in european indoor environments and associated health risk. Environ. Int..

[19] Ras M.R., Borrull F., Marcé R.M. (2009). Sampling and preconcentration techniques for determination of volatile organic compounds in air samples. TrAC Trends Anal. Chem..

[20] Zhang Y., Zhao J., Du T., Zhu Z., Zhang J., Liu Q. (2017). A gas sensor array for the simultaneous detection of multiple VOCs. Sci. Rep..

[21] Park J., Tabata H. (2021). Gas sensor array using a hybrid structure based on zeolite and oxide semiconductors for multiple bio-gas detection. ACS Omega.

[22] Qin P., Okur S., Li C., Chandresh A., Mutruc D., Hecht S., Heinke L. (2021). A photoprogrammable electronic nose with switchable selectivity for VOCs using MOF films. Chem. Sci..

[23] Yang Y., Huang H., Wang Y., Qiu F., Feng Y., Song X., Tang X., Zhang G., Liu W. (2018). A family of mixed-lanthanide metal–organic framework thermometers in a wide temperature range. Dalton Trans..

[24] Ding Y., Lu Y., Yu K., Wang S., Zhao D., Chen B. (2021). MOF-nanocomposite mixed-matrix membrane for dual-luminescence ratiometric temperature sensing. Adv. Opt. Mater..

[25] Zhou Y., Yan B. (2015). Ratiometric detection of temperature using responsive dual-emissive MOF hybrids. J. Mater. Chem. C.

[26] Xia T., Cui Y., Yang Y., Qian G. (2017). A luminescent ratiometric thermometer based on thermally coupled levels of a Dy-MOF. J. Mater. Chem. C.

[27] Wong K.-L., Law G.-L., Yang Y.-Y., Wong W.-T. (2006). A highly porous luminescent terbium–organic framework for reversible anion sensing. Adv. Mater..

[28] Yang J., Che J., Jiang X., Fan Y., Gao D., Bi J., Ning Z. (2022). A novel turn-on fluorescence probe based on Cu(II) functionalized metal-organic frameworks for visual detection of uric acid. Molecules.

[29] Che J., Jiang X., Fan Y., Li M., Zhang X., Gao D., Ning Z., Li H. (2022). A Novel Dual-Emission Fluorescence Probe Based on CDs and Eu^3+^ Functionalized UiO-66-(COOH)_2_ Hybrid for Visual Monitoring of Cu^2+^. Materials.

[30] Wang X.R., Wang X.Z., Du J., Huang Z., Liu Y.Y., Huo J.Z., Liu K., Ding B. (2019). Post-synthetic dual-emission rhodamine B@ZIF-365 hybrid material and enzymatic biosensor enzyme@ZIF-365: Ratiometric temperature sensing, biomolecule nicotinamide detection and sensing platform for lactose and Al^3+^. J. Solid State Chem..

[31] Li M., Dong C., Yang J., Yang T., Bai F., Ning Z., Gao D., Bi J. (2021). Solvothermal synthesis of La-based metal-organic frameworks and their color-tunable photoluminescence properties. J. Mater. Sci. Mater. Electron..

[32] Dong C.-L., Li M.-F., Yang T., Feng L., Ai Y.-W., Ning Z.-L., Liu M.-J., Lai X., Gao D.-J. (2021). Controllable synthesis of Tb-based metal–organic frameworks as an efficient fluorescent sensor for Cu^2+^ detection. Rare Metals.

[33] Ashwin B.C.M.A., Sivaraman G., Stalin T., Yuvakkumar R., Muthu Mareeswaran P. (2018). Selective and sensitive fluorescent sensor for Pd^2+^ using coumarin 460 for real-time and biological applications. J. Photochem. Photobiol. B.

[34] Feng L., Dong C., Li M., Li L., Jiang X., Gao R., Wang R., Zhang L., Ning Z., Gao D. (2020). Terbium-based metal-organic frameworks: Highly selective and fast respond sensor for styrene detection and construction of molecular logic gate. J. Hazard. Mater..

[35] Zhou Y., Zhang D., Zeng J., Gan N., Cuan J. (2018). A luminescent lanthanide-free MOF nanohybrid for highly sensitive ratiometric temperature sensing in physiological range. Talanta.

[36] Cadiau A., Brites C.D.S., Costa P.M.F.J., Ferreira R.A.S., Rocha J., Carlos L.D. (2013). Ratiometric nanothermometer based on an emissive Ln^3+^-organic framework. ACS Nano.

[37] Fan X., Freslon S., Daiguebonne C., Pollès L.L., Calvez G., Bernot K., Yi X., Huang G., Guillou O. (2015). A family of lanthanide-based coordination polymers with boronic acid as ligand. Inorg. Chem..

[38] Miyata K., Konno Y., Nakanishi T., Kobayashi A., Kato M., Fushimi K., Hasegawa Y. (2013). Chameleon luminophore for sensing temperatures: Control of metal-to-metal and energy back transfer in lanthanide coordination polymers. Angew. Chem. Int. Ed..

[39] Zhao S.-N., Li L.-J., Song X.-Z., Zhu M., Hao Z.-M., Meng X., Wu L.-L., Feng J., Song S.-Y., Wang C. (2015). Lanthanide ion codoped emitters for tailoring emission trajectory and temperature sensing. Adv. Funct. Mater..

[40] Meng X., Song S.-Y., Song X.-Z., Zhu M., Zhao S.-N., Wu L.-L., Zhang H.-J. (2014). A Eu/Tb-codoped coordination polymer luminescent thermometer. Inorg. Chem. Front..

