# Supplementary material for: A Novel Dye-Modified Metal–Organic Framework as a Bifunctional Fluorescent Probe for Visual Sensing for Styrene and Temperature
Source: Molecules. 2023 Jun 22;28(13):4919. doi: 10.3390/molecules28134919 (PMC10343389; doi:10.3390/molecules28134919)
Supplement: Supplementary file 1 [file molecules-28-04919-s001.zip › molecules-2457757-supplementary.pdf]

A Novel Dye-Modified Metal–Organic Framework as a Bifunctional Fluorescent Probe for Visual Sensing for Styrene and Temperature

Jie Yang [1], Chaojun Ren [2], Min Liu [1], Wenwei Li [1], Daojiang Gao [1], Hongda Li [3] and Zhanglei Ning [1,*]

1 College of Chemistry and Materials Science, Sichuan Normal University, Chengdu 610068, China; yangjie_deyouxiang@163.com (J.Y.); liumin200009@163.com (M.L.); liwenwen202202@163.com (W.L.); daojianggao@sicnu.edu.cn (D.G.)
2 Beijing Aerospace Propulsion Institute, Beijing 100076, China; rcj_ustb@163.com
3 Liuzhou Key Laboratory for New Energy Vehicle Power Lithium Battery, School of Electronic Engineering, Guangxi University of Science and Technology, Liuzhou 545006, China; hdli@gxust.edu.cn
* Correspondence: zlning@sicnu.edu.cn

Supporting information

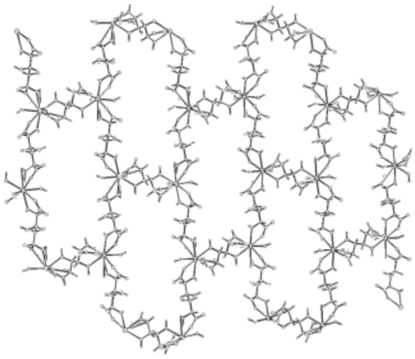

Figure S1 The diagram of the 2D network of Tb-MOFs [27].

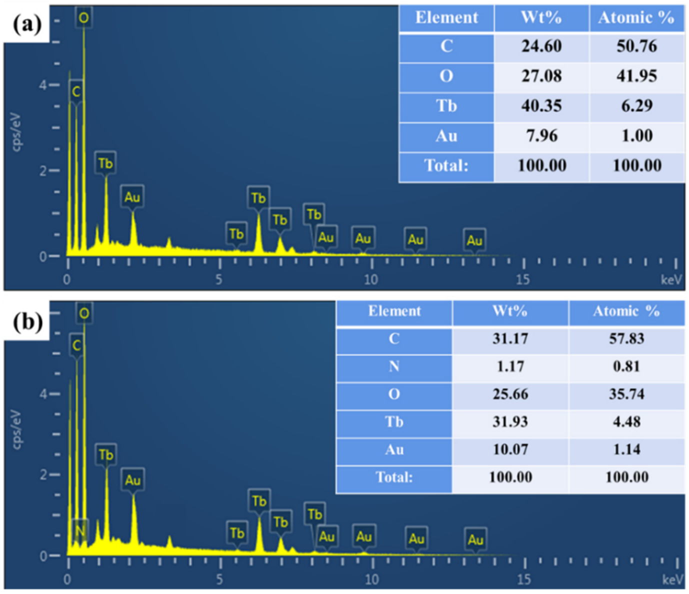

Figure S2 (a) EDX spectrums of Tb-MOFs; (b) EDX spectrums of C460@Tb-MOFs.

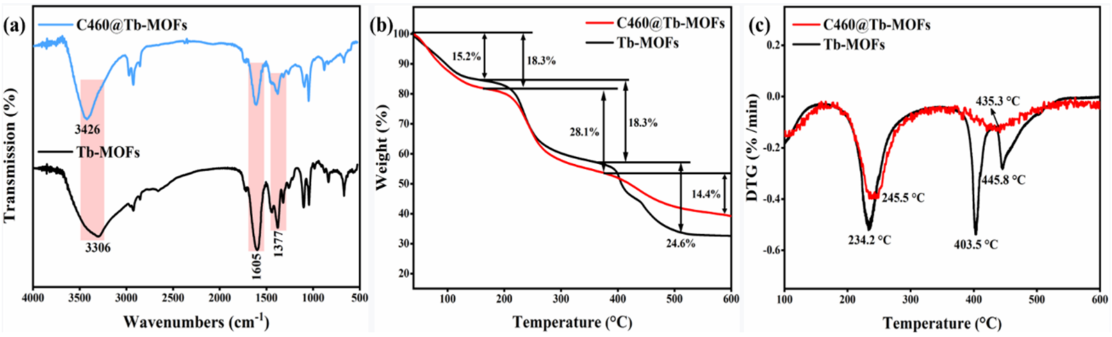

Figure S3 (a) FT-IR spectra of Tb-MOFs and C460@ Tb-MOFs; (b) The thermogravimetric curve of Tb-MOFs and C460@Tb-MOFs; (c) The DTG curve of Tb-MOFs and C460@Tb-MOFs.

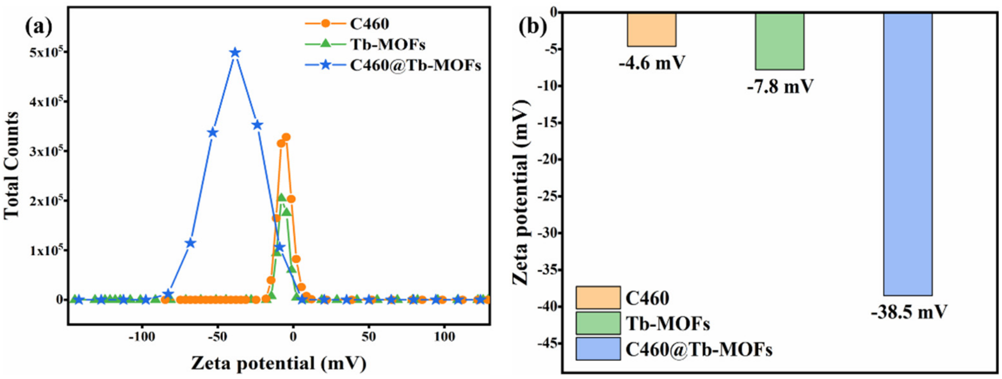

Figure S4 (a) Zeta potential plots of C460, Tb-MOFs and C460@Tb-MOFs; (b) Histograms of zeta potential values of C460, Tb-MOFs and C460@Tb-MOFs.

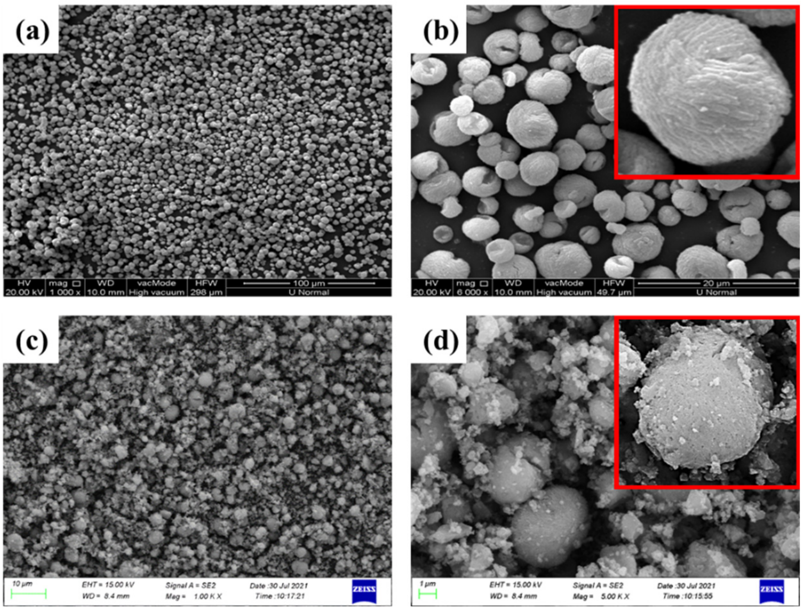

Figure S5 (a) SEM image of Tb-MOFs (low magnification); (b) SEM image of Tb-MOFs (high magnification), inset shows the details at this magnification; (c) SEM image of C460@Tb-MOFs (low magnification); (d) SEM image of C460@Tb-MOFs (high magnification), inset shows the details at this magnification.

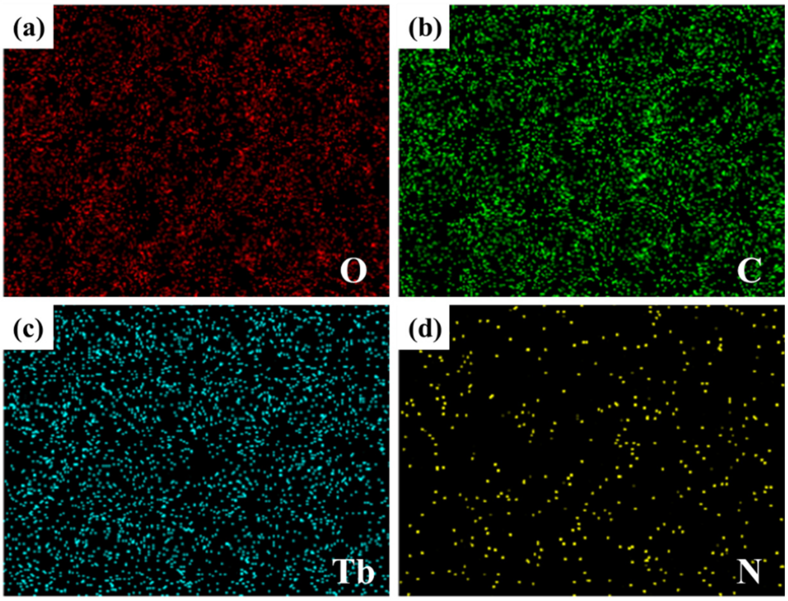

Figure S6 Elemental mapping images of C460@Tb-MOFs: (a) O element; (b) C element; (c) Tb element; (d) N element.

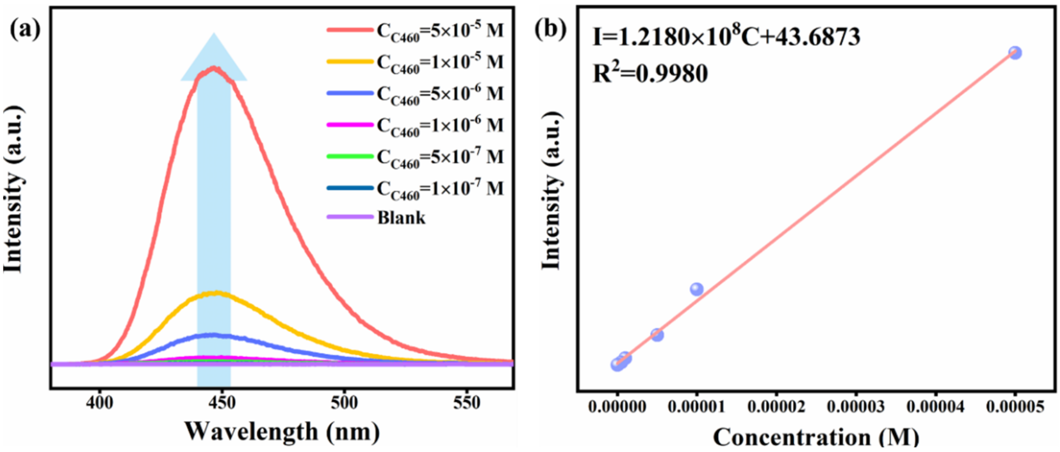

Figure S7 (a) Emission spectra of concentration gradient dye C460; (b) Fitted relationship between C460 luminescence intensity and concentration.

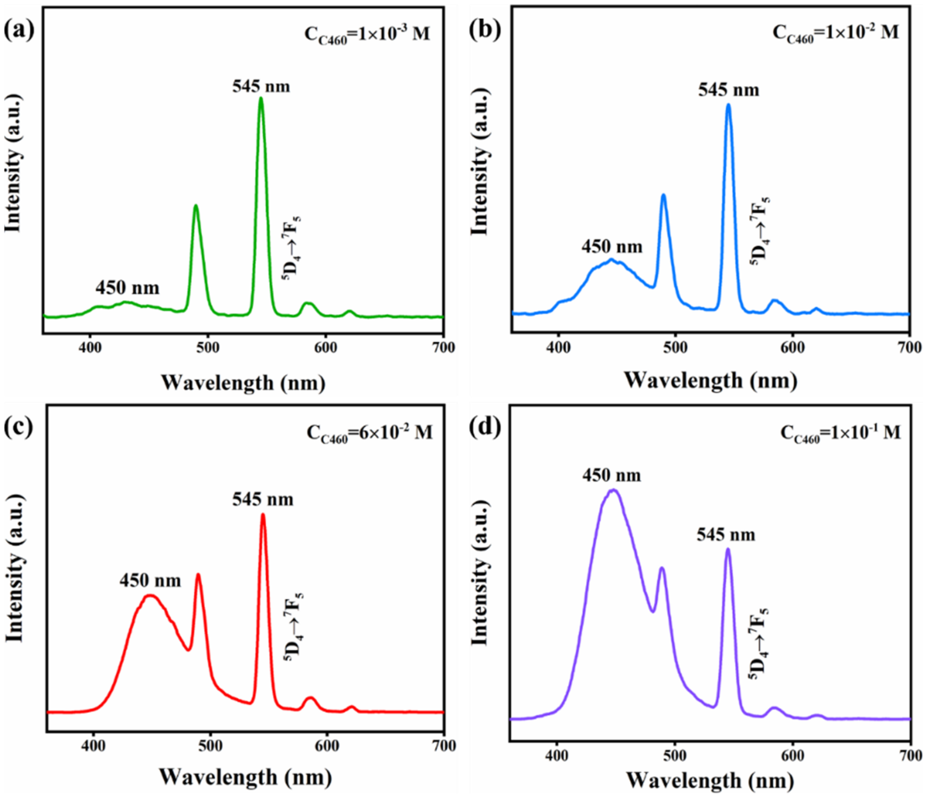

Figure S8 Emission spectra of C460@Tb-MOFs (different concentrations of dye): (a) The concentrations of C460 is 10^{-3} M with 0.004% loading; (b) The concentrations of C460 is 10^{-2} M with 0.034% loading; (c) The concentrations of C460 is 6×10^{-2} M with 0.49% loading; (d) The concentrations of C460 is 10^{-1} M with 0.67% loading.

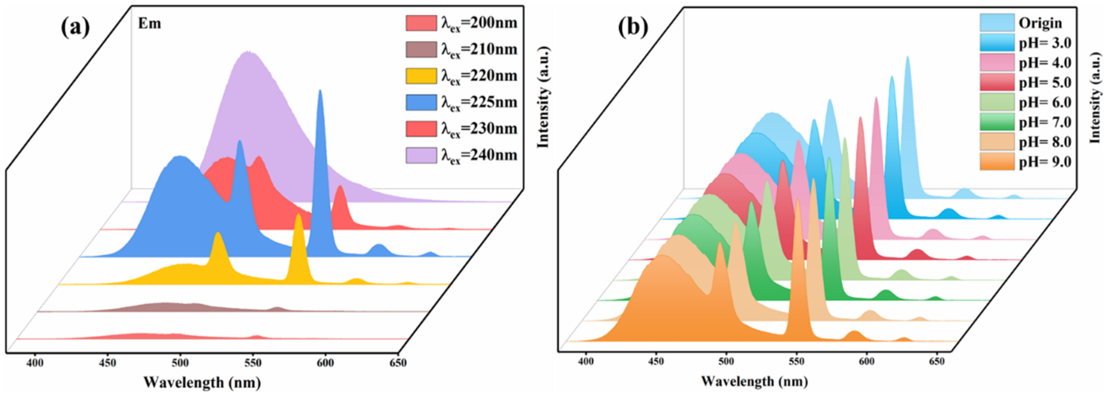

Figure S9 (a) Emission spectra of C460@Tb-MOFs at different excitation wavelengths; (b) Emission spectra of C460@Tb-MOFs in different pH environments.

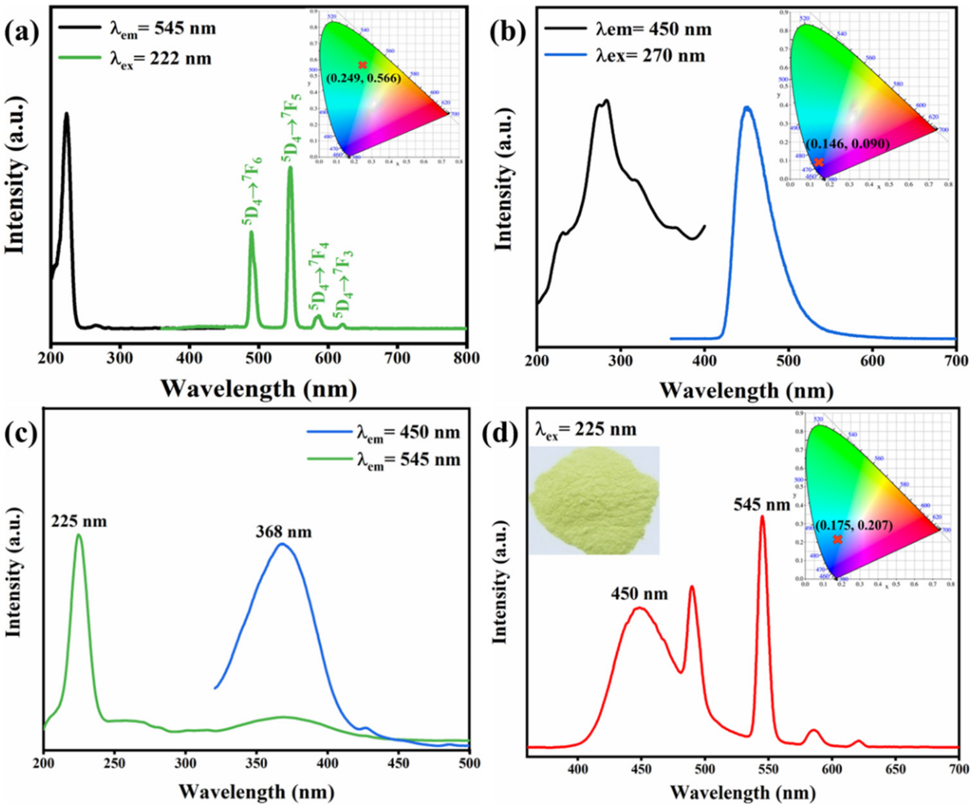

Figure S10 (a) The excitation and emission spectra of Tb-MOFs, inset is CIE chromaticity diagram for the Tb-MOFs; (b) The excitation and emission spectra of C460, inset is CIE chromaticity diagram for the C460; (c) Excitation spectra of C460@Tb-MOFs; (d) Emission spectra of C460@Tb-MOFs, inset is CIE chromaticity diagram for the C460@Tb-MOFs.

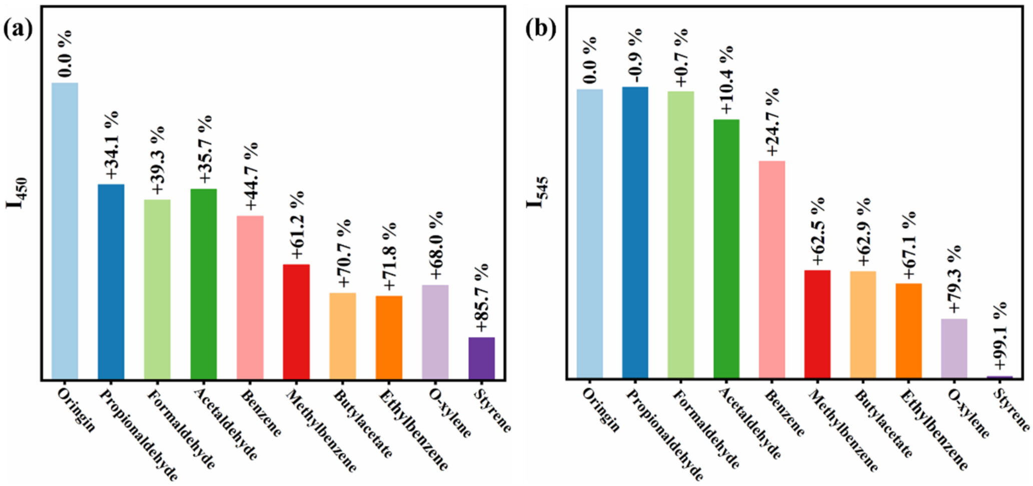

Figure S11 (a) The quenching rates of C460@Tb-MOFs at I_{450} for different VOCs solutions; (b) The quenching rates of C460@Tb-MOFs at I_{545} for different VOCs solutions.

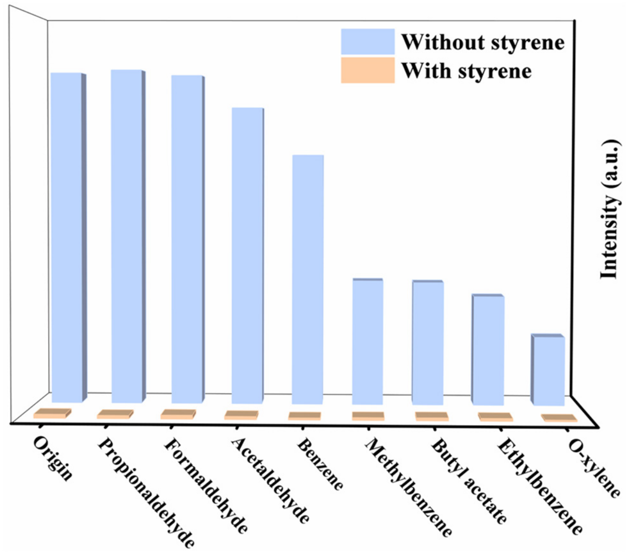

Figure S12 Anti-interference of C460@Tb-MOFs to styrene identification in the context of the presence of other interfering VOCs.

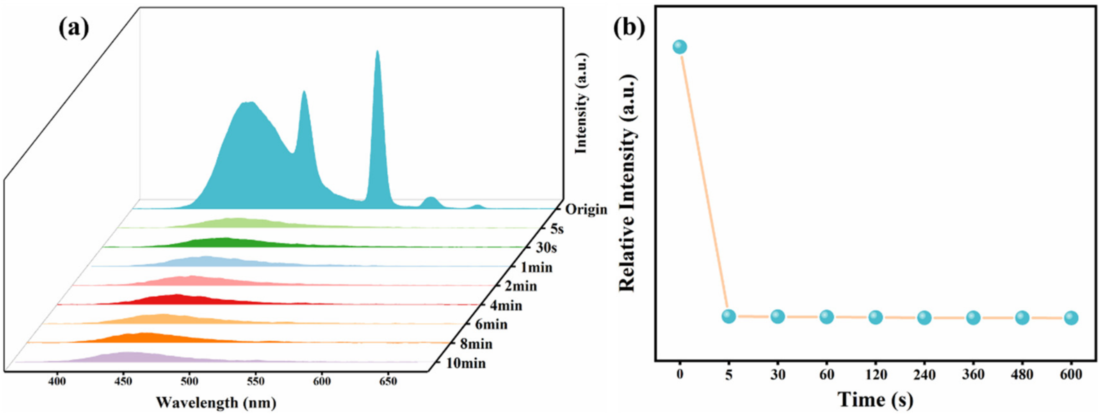

Figure S13 (a) Emission spectra of the time response of styrene detection by C460@Tb-MOFs; (b) Relative intensity ratio (I_{545}/I_{450}) versus time for styrene detection by C460@Tb-MOFs.

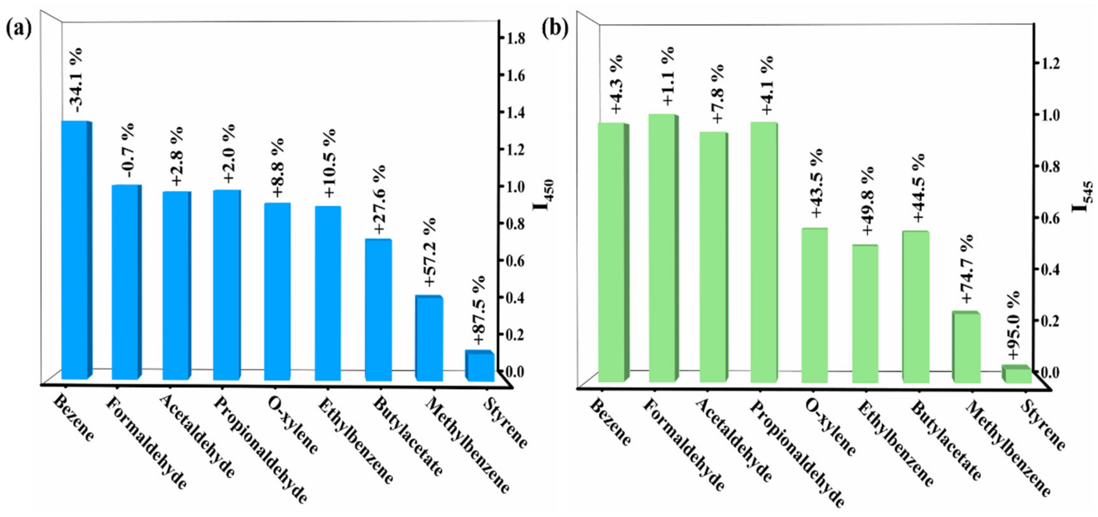

Figure S14 (a) The quenching rates of C460@Tb-MOFs at I_{450} for different VOCs gases; (b) The quenching rates of C460@Tb-MOFs at I_{545} for different VOCs gases.

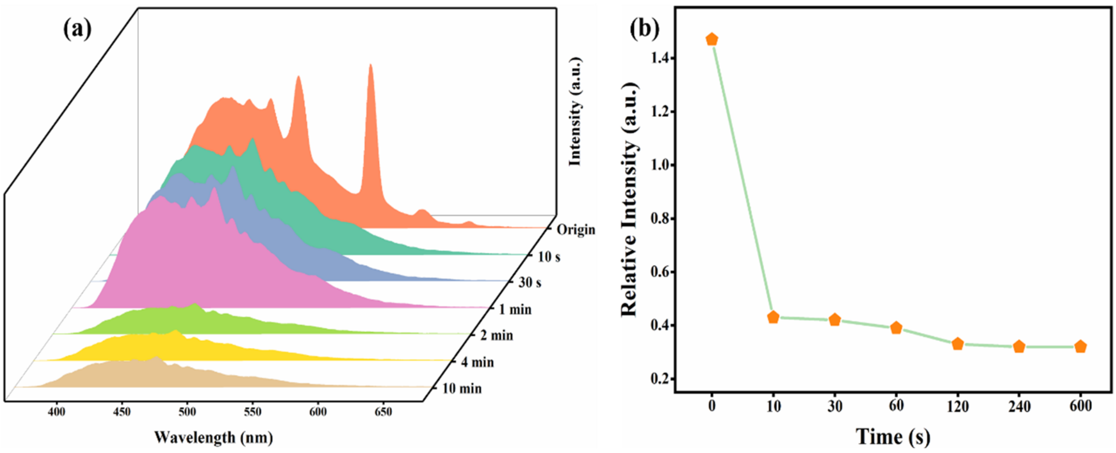

Figure S15 (a) Emission spectra of the time response of C460@Tb-MOFs silica gel plates for the detection of styrene gas; (b) The relative intensity ratio versus time for the detection of styrene gas by C460@Tb-MOFs silica gel plates.

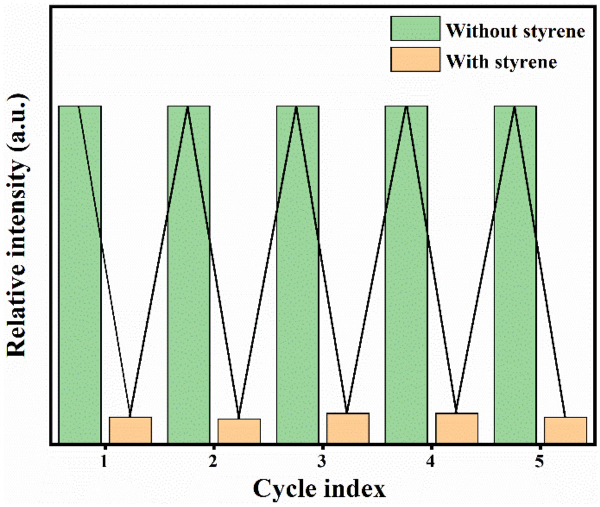

Figure S16 The histogram of relative fluorescence intensity of C460@Tb-MOFs silica gel plate after five cycles of styrene gas detection.
